# Macrophage Exposure to Polycyclic Aromatic Hydrocarbons From Wood Smoke Reduces the Ability to Control Growth of *Mycobacterium tuberculosis*

**DOI:** 10.3389/fmed.2018.00309

**Published:** 2018-11-13

**Authors:** Isabel Sada-Ovalle, Leslie Chávez-Galán, Luis Vasquez, Stepahnie Aldriguetti, Irma Rosas-Perez, Alejandra Ramiréz-Venegas, Rogelio Perez-Padilla, Luis Torre-Bouscoulet

**Affiliations:** ^1^Laboratorio de Inmunología Integrativa, Instituto Nacional de Enfermedades Respiratorias Ismael Cosío Villegas, Ciudad de México, Mexico; ^2^Departamento de Ciencias Ambientales, Universidad Nacional Autonoma de Mexico, Ciudad de Mexico, Mexico; ^3^Departmento de Tabaquismo, Instituto Nacional de Enfermedades Respiratorias Ismael Cosío Villegas, Ciudad de México, Mexico; ^4^Medica Sur, Clinic and Foundation, Mexico City, Mexico

**Keywords:** PAHs, macrophages, tuberculosis, mitochondrial disfunction, organic extracts

## Abstract

Use of solid fuels for cooking or home heating has been related to various diseases of the respiratory tract. Woodsmoke contains a mixture of carcinogenic polycyclic aromatic hydrocarbons (PAHs) and volatile organic compounds. Inhalation of these materials induces local and systemic changes in the immune system which may impair critical cell defense mechanisms; however, few studies have investigated the early effects that PAH exposures have on immune cells as macrophages. The aim of this study was to analyze if the pre-exposure to PAHs derived from woodsmoke deteriorates macrophage ability to control the intracellular growth of *Mycobacterium tuberculosis*. By using an *in vitro* experimental model, we analyzed the phenotype and some metabolic changes on THP-1 and monocyte-derived macrophages. Our results demonstrated that exposure to PAHs leads to cell activation and deteriorates mitochondrial function of the macrophage thus facilitating growth of *M. tuberculosis*.

## Introduction

Chronic exposure to wood smoke has a direct impact on lung health especially in women and children under 5 years old. According to the World Health Organization, “around 3 billion people cook and heat their homes using open fires and simple stoves burning biomass”^1^. Wood smoke particles (WSP) are very complex in their composition and have been associated with toxic compounds like polycyclic aromatic hydrocarbons (PAHs), carbon oxides, nitrogen oxides, metals, and endotoxins (1). All these compounds contribute to the development of lung inflammation as well as chronic immune activation (2). Several of the toxic compounds that have been identified in wood smoke have an inhalable size range with a mean aerodynamic diameter of <10 mm (PM10), so they can penetrate into the upper airways and the lung parenchyma, fine particles (PM2.5) are deposited in the alveoli through sedimentation and Brownian diffusion (3). PM1 can be translocated from different sites in the lung parenchyma through systemic circulation to several organs. There is a wide variation in the composition and size of the emissions when wood is burned, depending on the cooking practices and the other solid compounds that may be present (paper, leaves, dung) (4, 5).

Cooking is the most frequent activity contributing to indoor air pollution. According to Peréz-Padilla and coworkers, the majority of rural areas in Mexico burn wood in open fireplaces or rudimentary stoves, producing substantial emissions; which, in the presence of limited ventilation, induce very high levels of indoor pollution (6). Some studies have linked inhalation of wood smoke with an increased risk of pulmonary tuberculosis, but these studies are limited by several confounding factors (7). A conceivable molecular mechanism for this association would be, impairment of the respiratory innate response against intracellular bacteria (8). The systemic effects of acute wood smoke exposure have been studied in firefighters showing secretion of proinflammatory cytokines (9, 10). Despite epidemiological evidence, the specific mechanisms by which PAHs impair lung defense against intracellular bacteria are not well-understood. The objective in this study was to analyze how the functionality of the macrophage was affected when exposed to PAHs and how these alterations could impact their ability to restrict bacterial growth.

Therefore, we conducted a study where THP-1 macrophages as well as human monocyte-derived macrophages (MDM) were exposed to increasing concentrations of the organic fraction of WSP collected in Zacatlan, Puebla, a rural area near Mexico City and then infected with pathogenic *Mycobacterium tuberculosis* H37Rv (M.tb-H37Rv). We used monocytes as primary cells for the most critical experiments and THP-1 macrophages for those where many cells were needed. We assessed maturation and cell activation of exposed vs. unexposed macrophages, mitochondrial membrane potential, apoptosis/necrosis, ATP release, and cytokine production as well as intracellular bacterial growth. Our findings indicate that organic extract induced activation of macrophages is mediated, at least in part, by ATP release, and this pathway could be responsible for the increased production of ROS and cytokines by activated macrophages. Therefore, we propose that cell exposure to organic extracts induce a proinflammatory microenvironment where mitochondrial function of the macrophage is deteriorated making the cell more prone to die and unable to restrict bacterial growth of *M. tuberculosis*.

## Materials and methods

### Ethics statement

Blood specimens were acquired from buffy coats by the blood bank personnel at the National Institute of Respiratory Diseases (INER), Mexico City. The study was approved by the Institutional Review Board (IRB # B03-12) of the INER and was conducted following the principles stipulated by the Declaration of Helsinki.

### Wood smoke material

Particulate matter with an aerodynamic size of 10 μm (PM10) was collected using a high-volume sampler from Zacatlan, a rural area in Puebla, Mexico. This area is considered one of the hot-spots where almost all habitants use wood as fuel for home cooking and heating. Sample collection of PM10 was performed using a cellulose nitrate filter per day. Filters were maintained in the darkness at 4°C in a desiccator before processing.

### Analysis of polycyclic aromatic hydrocarbons

Filter extractions were performed with 10 mL of dichloromethane at 60°C for 10 min (twice) in an ultrasonic bath (WUC-D06H, WiseClean, Seoul, Korea). Extracts were filtered. Solvent was evaporated under a gentle nitrogen flow. A final volume of 1 mL was obtained. PAHs were analyzed by gas chromatography–quadrupole mass spectrometry (GC-MS; model 6890 plus/5973N, Agilent Technologies, CA, USA). DB35-MS capillary column (30 m in length × 0.25 mm internal diameter × 0.25 μm film thicknesses, J&W Scientific, USA) was used for PAH analysis. Helium (99.998%, Infra) was used as carrier gas at a flow rate of 1.2 mL min^−1^. One microliter of the sample was injected in splitless mode at 300°C. The oven temperature program was as follows: 40°C; hold for 1 min, first heating rate: 50°C min^−1^ to 110°C; hold for 0 min; second rate: 5°C min^−1^ to 303°C; hold for 0 min; third rate: 20°C min^−1^ to 335°C; and hold for 13 min. The transfer line, ion source, and quadrupole temperatures were 300, 230, and 150°C, respectively. The mass spectrometer was operated in electron ionization mode at 70 eV with selected ion monitoring.

### Determination of endotoxins by using the limulus amebocyte lysate colorimetric method

Endotoxin content was measured using the Limulus Amebocyte Lysate assay (LAL) according to the manufacturer's specifications (BioWhittaker, Walkersville, MD, USA) using Escherichia coli endotoxin as standard. Endotoxin results showed that PM10 collected displayed <0.01 UE/mg.

### THP-1 monocytes

The human monocyte cell line THP-1 (American Tissue Type Collection, Rockville, MD, USA) was grown in a humidified atmosphere at 37°C and 5% CO_2_ in RPMI-1640 medium with L-glutamine (2 mM, GIBCO, Grand Island, NY, USA), supplemented with 10% heat-inactivated fetal bovine serum (GIBCO, Grand Island, NY, USA), 10 mM HEPES, 1 mM pyruvic acid (Sigma-Aldrich, Steinheim, Germany), and penicillin/streptomycin (Sigma-Aldrich, Steinheim, Germany). THP-1 human monocytes were differentiated into macrophages by adding phorbol 12-myristate 13-acetate (PMA) (Sigma-Aldrich, Steinheim, Germany) (100 nM/mL) for 3 h at 37°C.

### Enrichment of monocytes

Peripheral blood mononuclear cells (PBMC) were isolated from buffy coats by standard Lymphoprep TM (Accurate Chemical-Scientific, Westbury, NY, USA) gradient centrifugation. Monocytes were enriched using positive selection via magnetic microbeads coated with antibodies to CD14 (Miltenyi Biotech). The CD14+ fraction was analyzed by flow cytometry to evaluate the purity of the cells. Purified cells were routinely 90–95% of the intended cell type. CD14+ enriched monocytes were plated at 1 × 10^5^ cells per well in 96 well plates (Costar, Ontario, Canada) with RPMI-1640 medium (GIBCO, Grand Island, NY, USA), supplemented by L-glutamine (2 mM; GIBCO, Grand Island, NY, USA), streptomycin-penicillin and 10% fetal bovine serum (FBS) (GIBCO, Grand Island, NY, USA). After a 7-day incubation period, viable cells were considered to be monocyte-derived macrophages (MDMs) based on their expression profile of CD14, CD68, and the mannose receptor (data not shown).

### Multiparametric flow cytometry analysis

To measure the expression of cell surface markers, 1 × 10^5^ THP-1 macrophages were stained and analyzed by flow cytometry. Briefly, cells were stained for 20 min at 4°C with fluorochrome-conjugated mAb to CD80, CD86, TLR2, TLR4, HLA-DR, IL-1 receptor 1, TNF-alpha receptor 1 and 2, DC-SIGN, and MMR (BioLegend, San Diego, CA). After incubation, cells were washed and re-suspended in staining buffer before flow cytometry. Flow cytometry acquisition was performed on a modified FACS Aria-II flow cytometer (Becton Dickinson, San Jose, CA) with 8 fluorescent parameter detection capabilities Compensation and analysis of data was performed using Flowjo software (Treestar). A list of the antibodies clones used can be found in Supplementary Table 1.

### Bacteria

*M. tuberculosis* H37Rv was obtained from the American Type Culture Collection (ATCC 25618, Manassas, VA). All experiments involving *M. tuberculosis* H37Rv strain were done in a BSL3 facility of the Institute according their regulations. Suspensions of M.tb-H37Rv were prepared in Middlebrook 7H9 broth medium supplemented with albumin-dextrose-catalase (ADC) (BD Bioscience, Franklin Lakes, NJ, USA) and 1% glycerol. After 21 days of incubation M.tb-H37Rv suspensions were harvested, aliquoted, and kept frozen at −86°C until use. The M.tb-H37Rv concentrations used in each experiment were confirmed from each thawed aliquot by a CFU assay of serial dilutions on 7H10 solid agar plates after 21-day incubations at 37°C. Frozen M.tb-H37Rv aliquots were thawed and centrifuged 5 min at 6,000 XG, and the resulting bacterial pellet was de-clumped by vortexing (5 min) with five sterile 3-mm glass beads suspended in RMPI-1640 medium with no antibiotics and enriched with 10% pooled human AB serum (Gemini Bio-Products, West Sacramento, CA, USA). The bacterial volume required to obtain the desired multiplicities of infection (MOI; 1:1) was calculated based on the CFU numbers known to be present in the M.tb-H37Rv suspension supernatants. The actual CFU numbers used for every *in vitro* infection were confirmed in each experiment.

### Macrophage exposure to organic extracts and *in vitro* infection with *M. tb*

MDM and THP-1 macrophages were grown in 24-well plates (5 × 10^5^ cells/well). The organic extract stock suspension (1 μg/μl) was prepared in complete RPMI-1640 medium. Cells were exposed to organic extracts for 24 h at the following concentrations: 1, 5, 10, and 20 μg/ml. Next, cells were washed twice with complete RPMI-1640 medium without antibiotics and then infected with M.tb-H37Rv at a multiplicity of infection of 1:1 for an additional 2 h. To remove all the extracellular bacteria cells were washed twice with PBS. Macrophages were then incubated for an additional 72 h (37°C and 5% CO2). At day 4 post-infection, cells were lysed with a 0.1% solution of saponin for 5 min and the bacteria were enumerated by plating serial dilutions of cell lysates on Middlebrook 7H10 agar plates. Colonies were counted after 21 days.

### Effect of organic extracts on mitochondrial membrane potential and cell death

MDM and THP-1 macrophages were grown in 24-well plates (5 × 10^5^ cells/well). Cells were exposed to organic extracts for 24 h at the following concentrations: 1, 5, and 10 μg/ml. Next, cells were washed twice with complete RPMI-1640 medium, and MitoTracker Red CM-H_2_XROS (500 nM) was added, and the incubation was continued for 40 min in the dark. Cells were washed twice with PBS and then analyzed with FlowJo (Tree Star, Inc., Ashland, OR). Typically, 100,000 events were acquired. Unexposed cells treated for 4 h with 200 ng/ml staurosporine (Sigma-Aldrich) were included as a positive apoptosis control. Apoptotic changes were identified by staining macrophages with FITC-conjugated annexin V according to the manufacturer instructions (R&D Systems). Specific binding of annexin V was achieved by incubating 10^6^ macrophages in 60 μl of binding buffer with annexin V for 15 min at 4°C. To differentiate between early apoptosis and necrosis, macrophages were simultaneously stained with propidium iodide (PI) before analysis. The binding of annexin V and PI to the cells was also measured by flow cytometry (FACSAria II; BD Biosciences). At least 10,000 cells were counted in each sample and a gate based on forward and side scatters was set to exclude cell debris.

### Determination of cell viability

The cytotoxic effect onTHP-1 and MDM macrophages following organic extracts exposure were assessed by staining the cells with the crystal violet dye. We seeded triplicate wells for each experimental condition. After the incubation period, methanol (methyl alcohol 99.8%—Sigma-Aldrich Co., St. Louis, MO, USA) was added to each well for 10 min. After cell fixation 100 μl 0.2% crystal violet was added for 15 min. Crystal violet was discarded, and plates were washed with distilled water. Plates were left to dry for 18 h. Triton X-100 (0.2%) (200 μl) was added to the wells and samples were incubated for 30 min at room temperature. Meanwhile, crystal violet was released from the cells into the supernatant. After incubation, 200 μl of the supernatant were transferred to a new 96 well plate. Absorbance was read using a 570 nm on SoftMax Pro ELISA analysis software (Molecular Devices). Calculation of cell viability was based on the measurements of cellular content of DNA (crystal violet binding) in the wells for each experimental condition and were calculated according the following formula: as the % control following formula: % cell viability = OD of experimental sample—background/mean OD control value—background X 100. In all experiments, the mean absorbance of the control wells, where the cells did not receive any treatment, was considered as 100% physiological cell response.

### Determination of extracellular ATP concentration

ATP levels in the cell culture medium were analyzed using the Bioluminescence Molecular Probes' ATP Determination Kit following the manufacturer's instructions. LPS (10 μg/ml) (Sigma-Aldrich) stimulated cells were included as a positive control. Briefly, 50 μL of each sample were mixed with 50 μL of the standard reaction solution, incubated at room temperature for 30 min, and then fluorescence was measured on a SpectraMax plate reader (Ex/Em = 535/587 nm) (Molecular device).

### Reactive oxygen species production

The production of ROS was measured by using the membrane permeable 2′-7′-dichlorofluorescin diacetate (H_2_DCFDA; Life Technologies Inc., Catalog No. D-399), which is oxidized to fluorescent 2′,7′-dichlorofluorescein (DCF) by ROS production following cleavage of its acetate groups by intracellular esterases. Exposed and unexposed THP-1 macrophages were incubated in sterile round-bottom polypropylene tubes with 10 μM H_2_DCFDA dissolved in DMSO for 30 min at 37°C, after which DCF MFI was measured by flow cytometry. IFN-γ (50 U/ml) (Genzyme) stimulated cells were included as a positive apoptosis control. After gating away cellular debris, the mean fluorescence intensity of exposed and unexposed cells was recorded.

### Determination of cytokines

Collected cell culture supernatants from the *M. tb*-infected THP-1 macrophages or MDMs were passed through a 0.2 μM filter to remove any bacteria. Supernatants were assayed for cytokines by a sandwich ELISA, which was conducted following the manufacturer's instructions with absorbance recorded at 405 nm on SoftMax Pro ELISA analysis software (Molecular Devices). The IFN-γ, IL-1β, and TNF-α in the culture supernatants were quantified by comparison with the appropriate recombinant standard (purchased from R&D Systems).

### Statistical analysis

Results are expressed as means ± SD. Data were analyzed by one-way ANOVA (with 95% confidence intervals) and Dunnett's posttest (for comparison against a single control) or unpaired Student's *t*-test. Analysis was performed using GraphPad Prism software (GraphPad Software, Inc., San Diego, CA).

## Results

### Chemical composition of WSP

**Figure 1:**
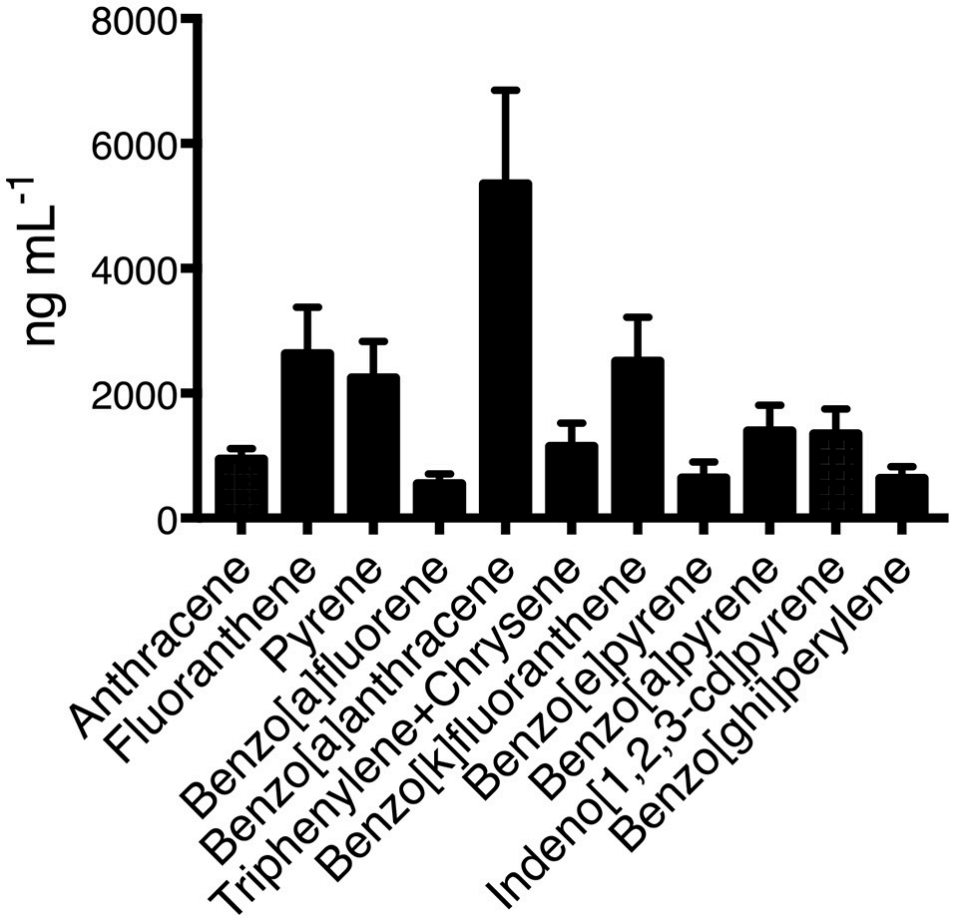
Concentrations of all analyzed PAHs present in the organic fraction of the wood smoke particles. Three independent samples were obtained and pooled from Zacatlan, Puebla. Means and standard deviations are shown.

The collected cellulose filters contained multiple carbon aggregates which had adsorbed PAHs, aldehydes, and aliphatic hydrocarbons. All the organic material obtained from the filters was pooled into a one single batch. The GC-MS analysis of the pooled material identified 11 different PAHs (Figure 1) with benzo-a-anthracene, fluoranthene, and being benzo-a-fluoranthene the most abundant. Other PAHs were also present in PM10 samples in very small concentrations. A representative chromatogram is shown as Supplementary Figure 1.

### Expression of cell surface receptors

**Figure 2:**
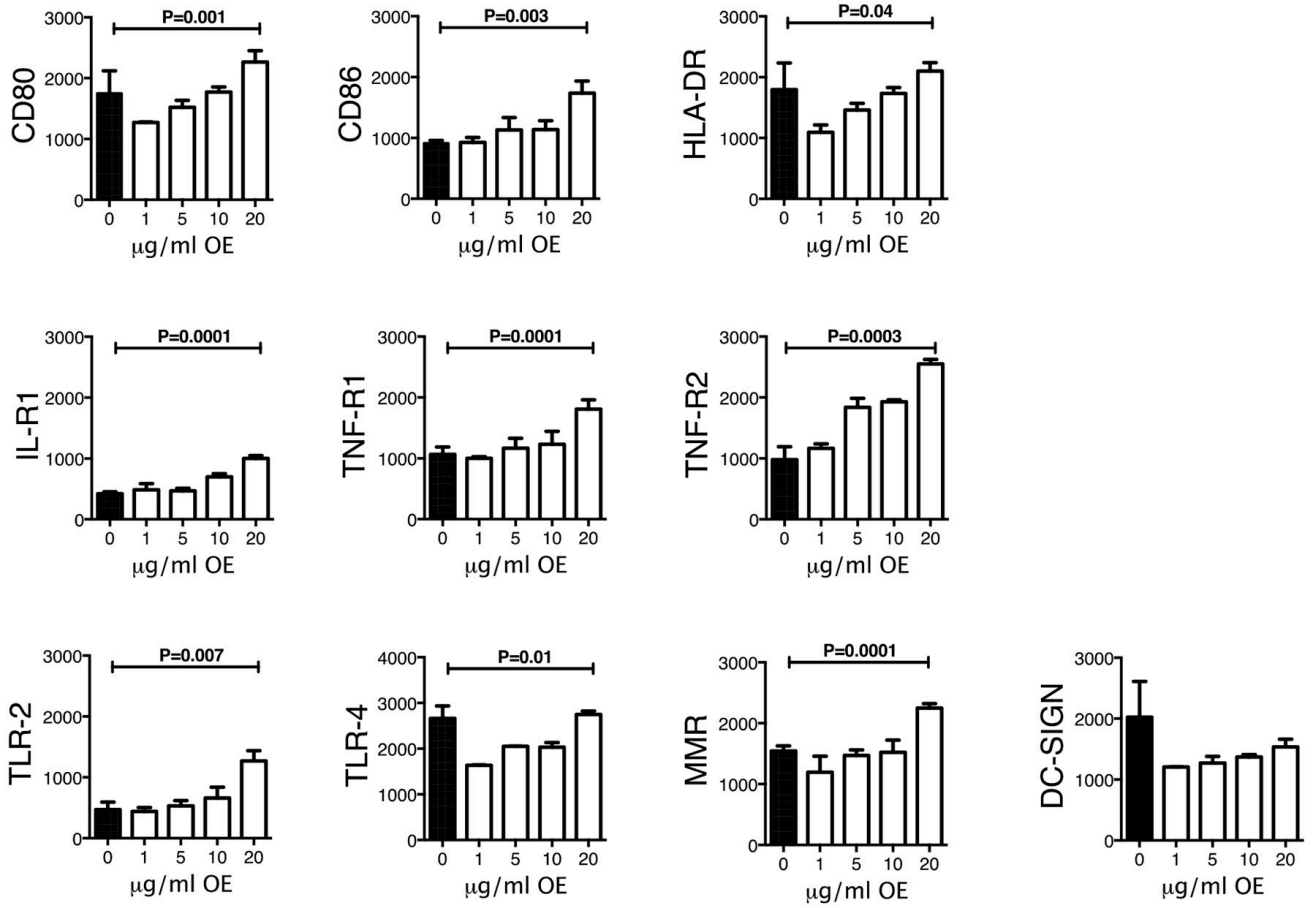
Expression of activation and maturation markers on THP-1 macrophages exposed to increasing concentrations of organic extracts. Cells were exposed to organic extracts for 24 h at the following concentrations: 1, 5, 10, and 20 μg/ml, then the mean fluorescence intensity (MFI, arbitrary units) of the different markers in macrophages was analyzed as indicated. Ten independent experiments were included. Bars show mean values and SD. The statistical analyses were performed using ANOVA and Dunnett's *post-hoc* test compared to untreated macrophages (black bar). *P*-values are indicated in each graph.

To identify whether phenotypic changes were induced in THP-1 macrophages exposed to increasing concentrations of organic extracts; CD80, CD86, TLR2, TLR4, HLA-DR, IL-1 receptor 1, TNF-alpha receptor 1 and 2, and MMR receptors were analyzed by multiparametric flow cytometry. As shown in Figure 2 the MFI of all activation and maturation markers except for DC-SIGN was gradually increased in a dose-dependent manner (*P* < 0.01) suggesting that maturation and cell activation had taken place.

### Organic extracts contributes to reduce macrophage ability to control *M. tuberculosis* growth

**Figure 3:**
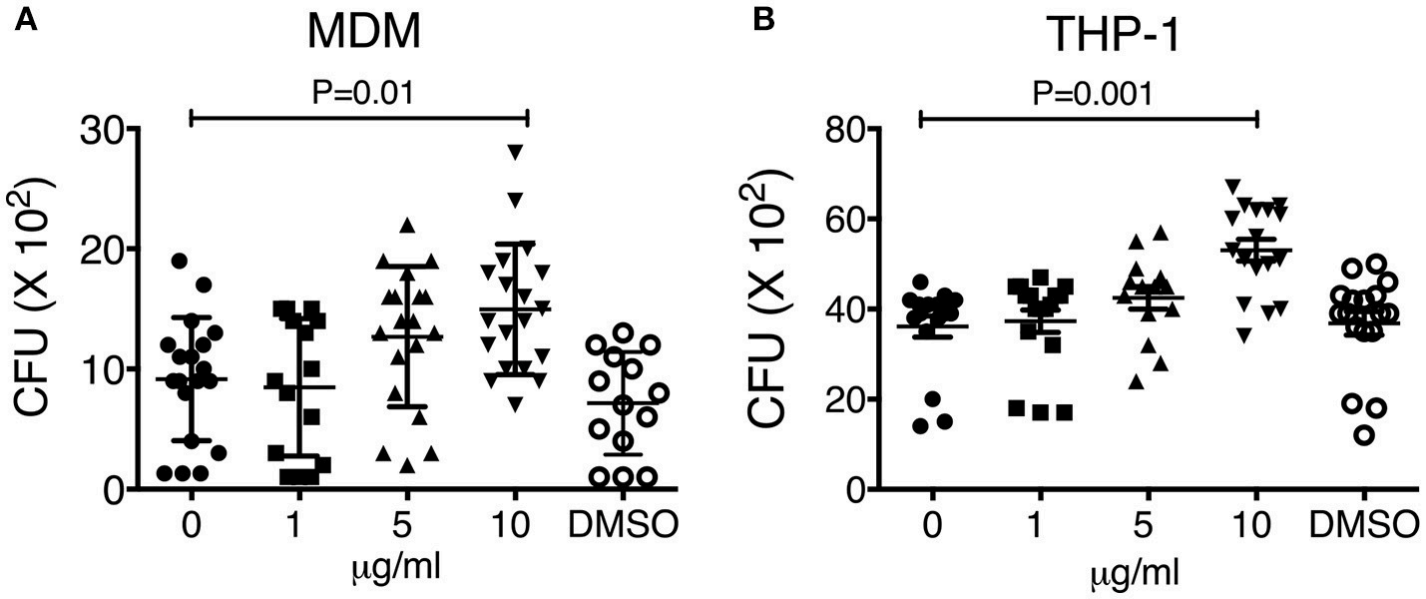
Macrophage exposure to organic extracts reduces macrophage ability to restrict the growth of *Mycobacterium tuberculosis*. MDM **(A)** and THP-1 **(B)** macrophages were stimulated with increasing concentrations of organic extracts (1, 5, and 10 μg/ml) during 24 h and then infected with M.tb-H37Rv (MOI 1). The negative control received a comparable amount of DMSO. On day 4th post-infection CFU were enumerated. Mean ± SD are represented form ten independent experiments. The statistical analyses were performed using ANOVA and Dunnett's *post-hoc* test compared to untreated macrophages. *P*-values are indicated in each graph.

We previously demonstrated that macrophage exposure to crystalline silica particles promotes necrosis and reduced host resistance against tuberculosis infection (11). The toxicological effects of PAHs have been well-documented in the last years. Oxidative stress and inflammation are the main pathways being affected after organic extract exposure, therefore, we hypothesized that an early exposure to this material may result in cell toxicity and deterioration of the macrophage mechanisms responsible for host defense against *M. tuberculosis*. To test this hypothesis, we exposed during 24 h THP-1 and MDM macrophages to 1, 5, 10, and 20 μg of organic extracts. Next, cells were infected for 2 h with M.tb-H37Rv with an MOI of 1:1 (bacteria/macrophage) and 3 days later cells were lysed to analyze bacterial growth. Our results showed that pre-exposure to organic extracts reduced macrophage capacity to restrict intracellular bacterial growth. The reduction of bacterial growth was dose-dependent in both, THP-1 and MDM macrophages (*P* < 0.01) (Figure 3).

### Organic extracts disrupt MMP

**Figure 4:**
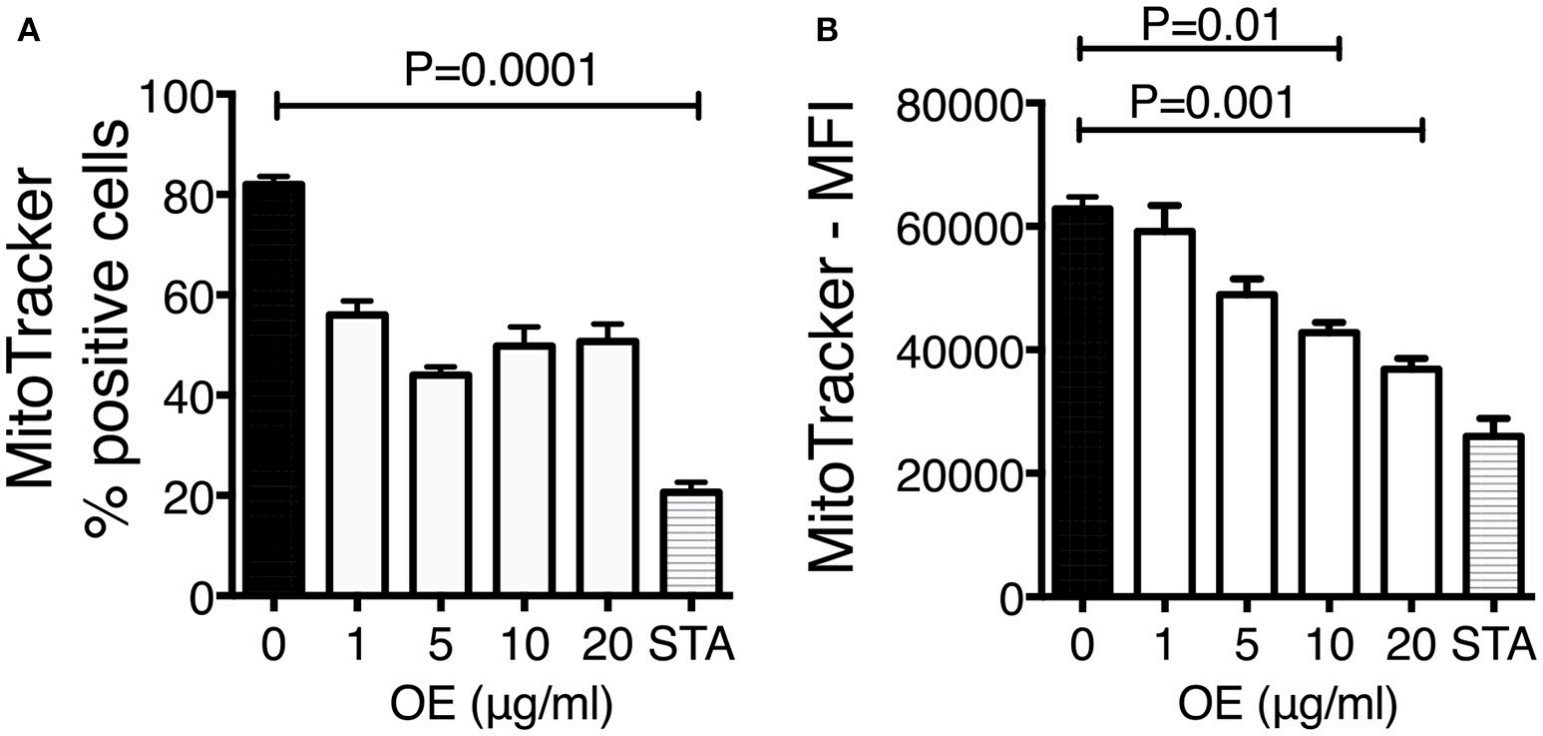
Organic extracts induce mitochondrial deterioration. THP-1 macrophages were stimulated with organic extracts (1, 5, 10, and 20 μg/ml) and incubated during 24 h, then the mitochondrial membrane potential was analyzed. Unexposed cells treated for 4 h with 200 ng/ml staurosporine (Sigma-Aldrich) were included as a positive control. Frequency **(A)** and mean fluorescence intensity (MFI) **(B)** of MitoTracker positive cells are shown from ten independent experiments. Mean SD are represented. The statistical analyses were performed using ANOVA and Dunnett's *post-hoc* test compared to untreated macrophages. *P*-values are indicated in each graph.

It has been demonstrated that PAHs from WSP are cytotoxic; however, the identification of the immune mechanisms and the physiologic impact underlying this phenomenon are not completely elucidated. We have demonstrated that several PAHs including benzo(a)pyrene were present in the organic extracts material, and it is known that these compounds can lead to mitochondrial dysfunction and cell death. To investigate whether mitochondrial dysfunction was involved as a potential mechanism underlying the increased bacterial growth previously observed, MMP levels and phosphatidylserine externalization were measured on THP-1 macrophages exposed during 24 h to increasing concentrations of organic extracts (1, 5, 10, and 20 μg). When the frequency of MitoTracker positive cells was analyzed we found that it was decreased with all organic extracts concentrations, but this reduction was not statically significant; however, when the mean fluorescence intensity was analyzed we were able to see a significant reduction of the MMP (*P* = 0.001). MMP was significantly impaired in all concentrations, with the maximum impairment measured with the highest concentration of organic extracts (Figure 4).

**Figure 5:**
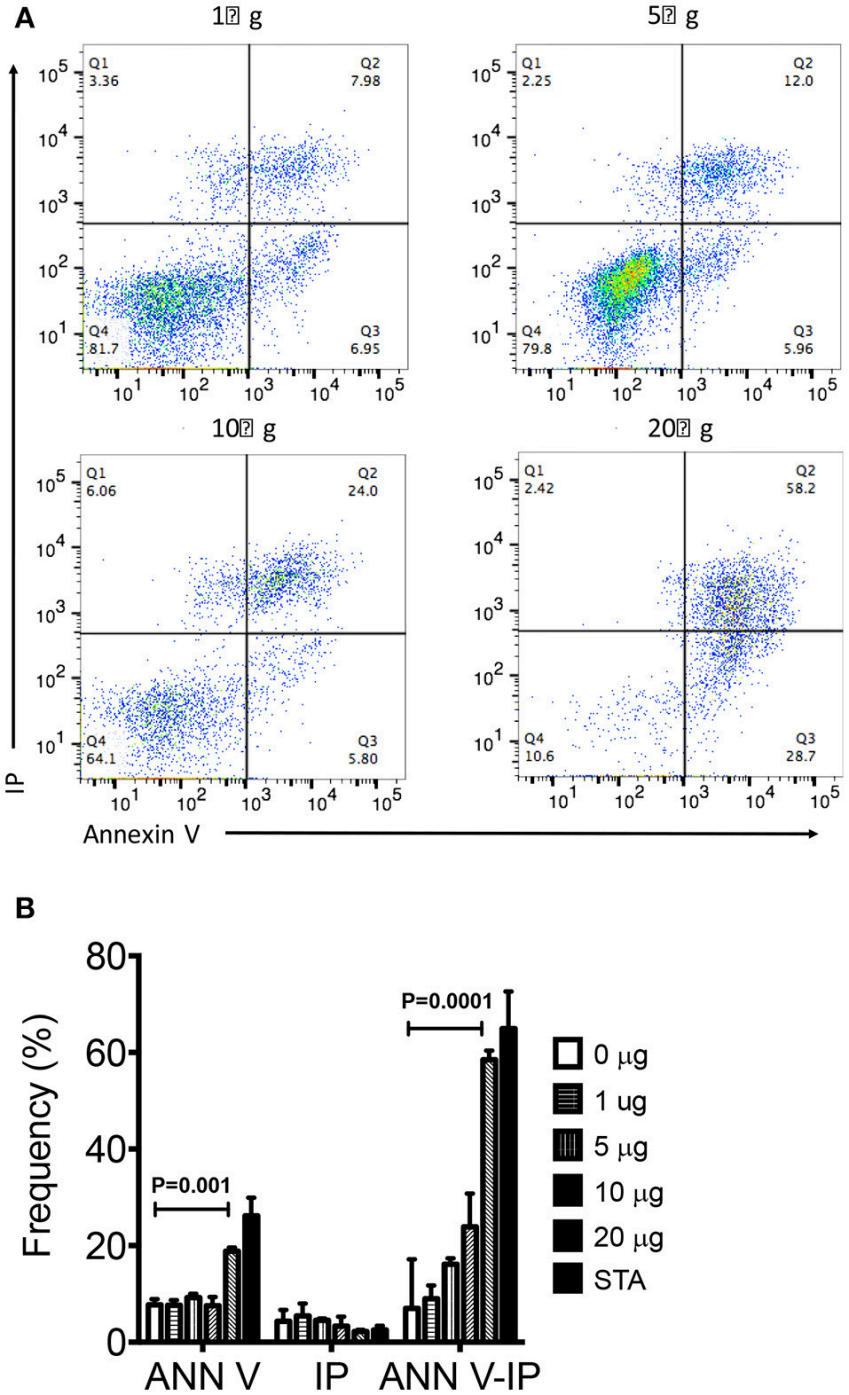
Organic Extracts promote macrophage cell death. THP-1 macrophages were stimulated with increasing concentrations of organic extracts (1, 5, 10, and 20 μg/ml) during 24 h; then, the percentage (%) of apoptotic and necrotic macrophages was analyzed. **(A)** A group of dot plots of apoptotic (annexin V+) and necrotic (annexin V+ IP+) macrophages are shown from a representative experiment, **(B)** Percentage of annexin V+, IP+, and annexin V+IP+ macrophages exposed to increasing concentrations of organic extracts are shown. The gate used to define annexin V and IP positive cells was set using the fluorescence minus one (FMO) control. Cells stimulated with 200 ng/ml staurosporine (Sigma-Aldrich) were included as a positive apoptosis control. Five independent experiments were included. Bars show mean values and SD. The statistical analyses were performed using ANOVA and Dunnett's *post-hoc* test compared to untreated macrophages (black bar). *P*-values are indicated in each graph.

We next assessed if organic extracts were able to induce not only mitochondrial deterioration but cell death. THP-1 macrophages were exposed to increasing concentration of organic extracts and then stained with annexin V and IP. The analysis of apoptotic (annexin V positive cells) and necrotic (annexin V + IP positive cells) cells showed that organic extracts induced cell death in a dose dependent manner (Figures 5A, B). From these experiments, we concluded that apoptosis of exposed-macrophages correlated with the loss of MMP. These results support our hypothesis that organic extracts deteriorate cell metabolism leading to cell death.

### Organic extracts induces cell death

**Figure 6:**
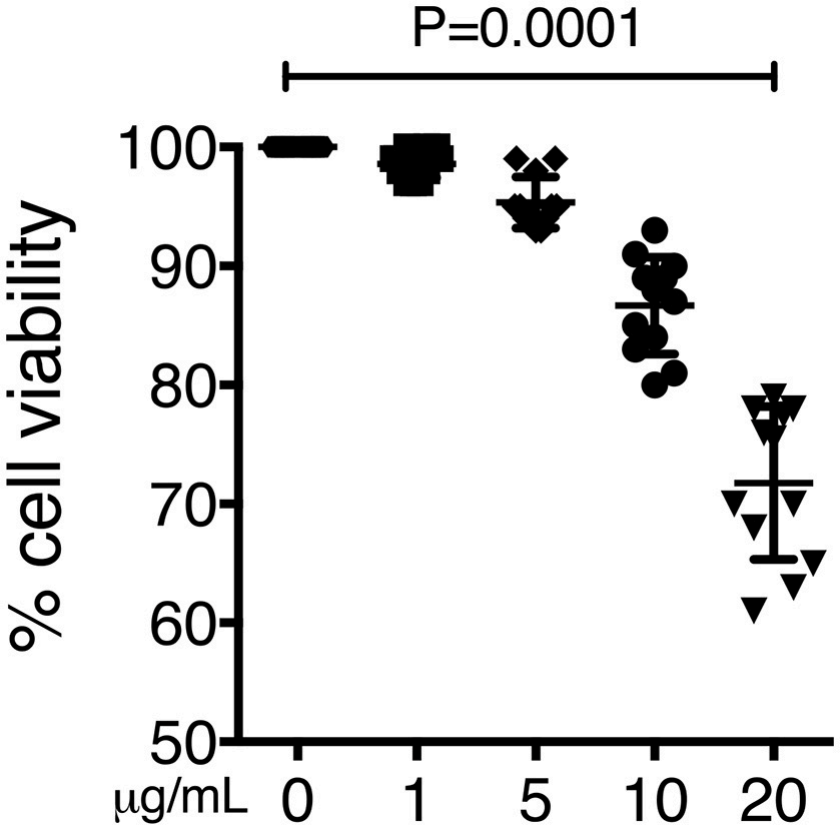
Cell death induced by organic extract exposure. THP-1 macrophages were exposed to increasing concentrations of organic extracts (1, 5, and 10 μg/ml) for 24 h. Cell viability was analyzed by staining the cells with the crystal violet dye. Mean ± SD are represented form ten independent experiments. The statistical analyses were performed using ANOVA and Dunnett's *post-hoc* test compared to untreated macrophages. *P*-values are indicated in each graph.

To confirm our previous results, we also screened cell viability by doing the crystal violet staining under the same experimental conditions. After the incubation period cells exhibited statistically lower cell viability in a dose dependent manner than the respective negative controls (*P* < 0.001) (Figure 6).

### Macrophage oxidative stress

**Figure 7:**
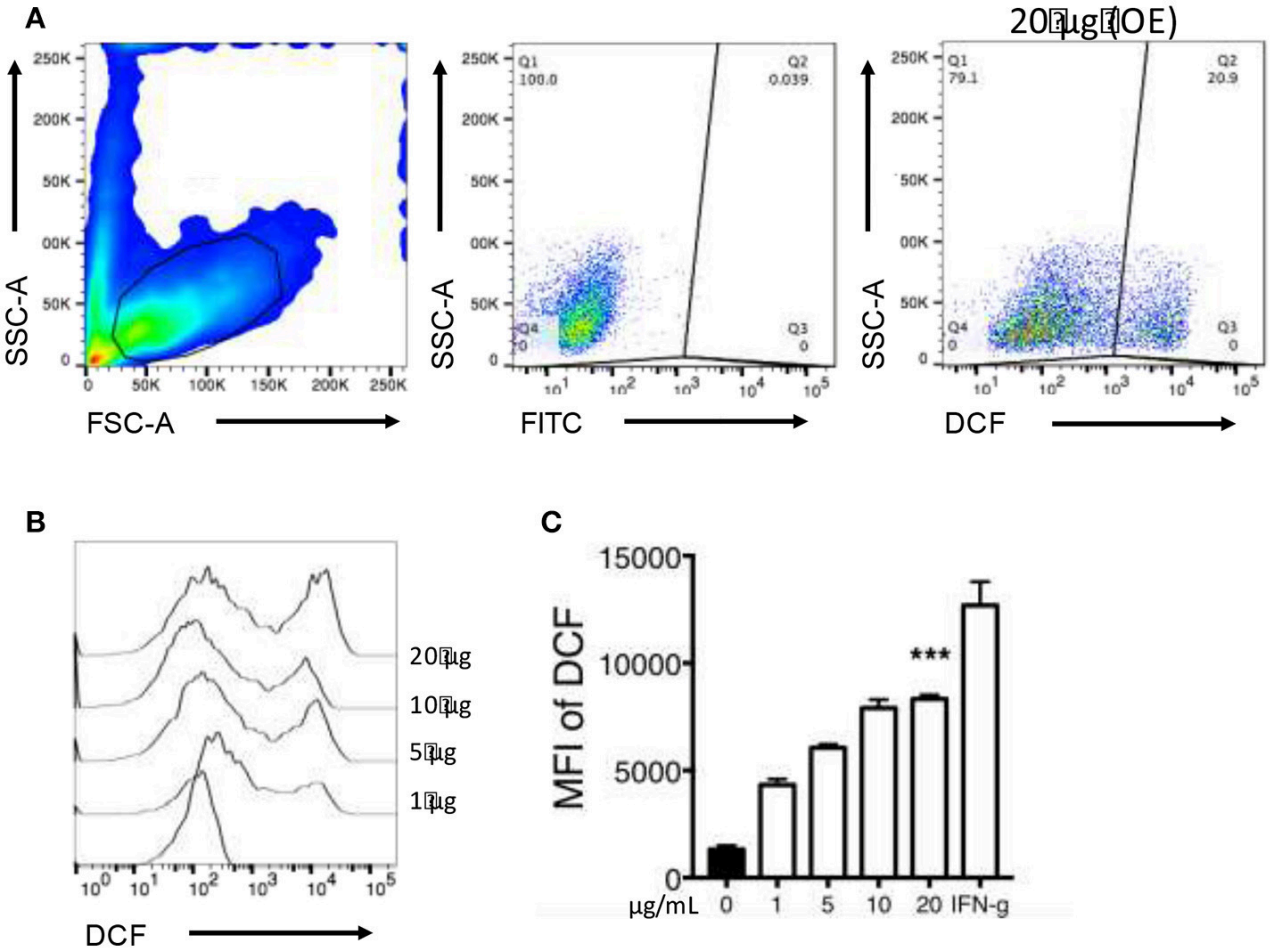
Organic Extracts activates oxidative stress in exposed macrophages. 2′,7′ dichlorodihydrofluorescein diacetate (DCF) fluorescence was used as an indicator of ROS production. THP-1 macrophages were stimulated with organic extracts and incubated for the indicated times, then the mean fluorescence intensity (MFI) of DCF was analyzed. From the FSC-A vs. SSC-C dot plot, the live macrophage population was analyzed. **(A)** ROS production was identified based on DCF expression. A dot plot from a representative experiment using the highest concentration of organic extracts is shown, **(B)** A histogram comparing ROS production in expose macrophages is shown, **(C)** ROS production is shown from five independent experiments. IFN-γ 50 (U/ml) (Genzyme) stimulated cells were included as a positive control. Bars show mean values and SD. The statistical analyses were performed using ANOVA and Dunnett's *post-hoc* test compared to untreated macrophages (black bar). ^***^P < 0.001.

Macrophage exposure to organic extracts induce ROS production (Figure 7A). The levels of ROS produced after organic extracts exposure were increased in a dose dependent manner compared with unexposed macrophages. However, only the highest concentration (20 μg/mL) induced a statistically significant ROS production (Figures 7B,C). Importantly, these results confirmed that although macrophages were exposed for 24 h only; the oxidative stress pathway was activated but probably not sufficiently to restrict bacterial growth of *M. tuberculosis*.

### Atp levels are dysregulated on macrophages exposed to organic extracts

**Figure 8:**
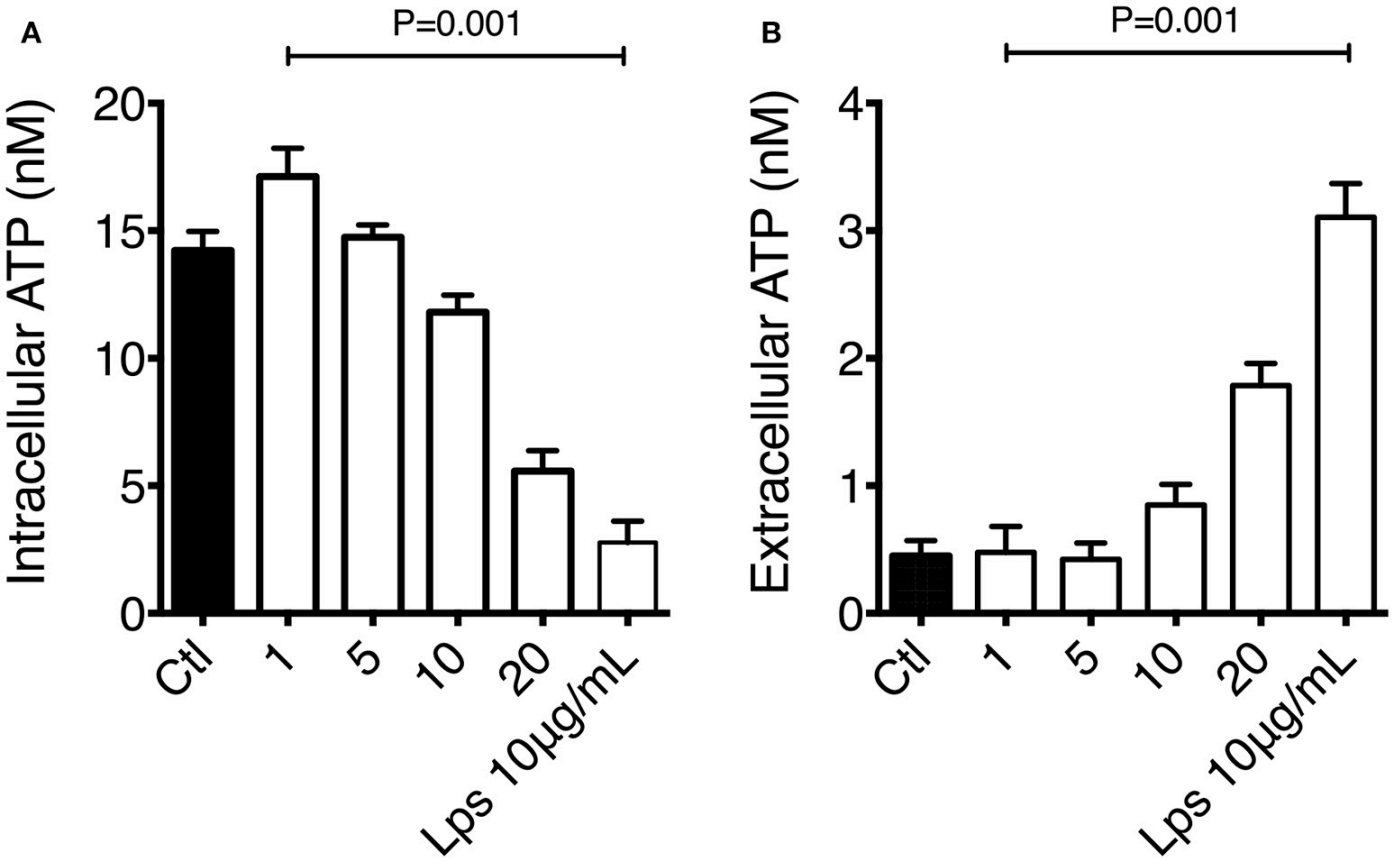
Macrophage exposure to organic extracts leads to ATP depletion. THP-1 macrophages were exposed to organic extracts (1, 5, and 10 μg/ml) for 24 h. Intracellular **(A)** and extracellular **(B)** levels of ATP were assessed by using the luciferin-luciferase assay. Concentration was expressed as nM. LPS 10 μg/ml (Sigma-Aldrich) stimulated cells were included as a positive control. Mean ± SD are represented form ten independent experiments. The statistical analyses were performed using ANOVA and Dunnett's *post-hoc* test compared to untreated macrophages (black bar).

It has been previously described that intracellular ATP levels are preserved by the mitochondrial membrane potential, and changes in ATP concentration can be stimuli to induce IL-1β production and secretion. We had previously demonstrated that macrophage exposure to organic extracts disrupted MMP. We confirmed the mitochondrial dysfunction by measuring the ATP secretion on macrophages exposed for 4 h to organic extracts. Our results have shown that intracellular ATP levels declined in a dose-dependent manner and increased in the same proportion in the culture supernatant (Figures 8A,B). ATP secretion by stressed or activated monocytes and macrophages is one of the stimuli that have been proposed to activate the NLRP3 inflammasome leading to IL-1β production. We hypothesized that this inflammasome activation is induced when macrophages are exposed to 1 and 5 μg/ml of organic extracts, then cells go into apoptosis and necrosis probably secondary to ATP depletion.

### Macrophage exposed to organic extracts secrete proinflammatory cytokines

**Figure 9:**
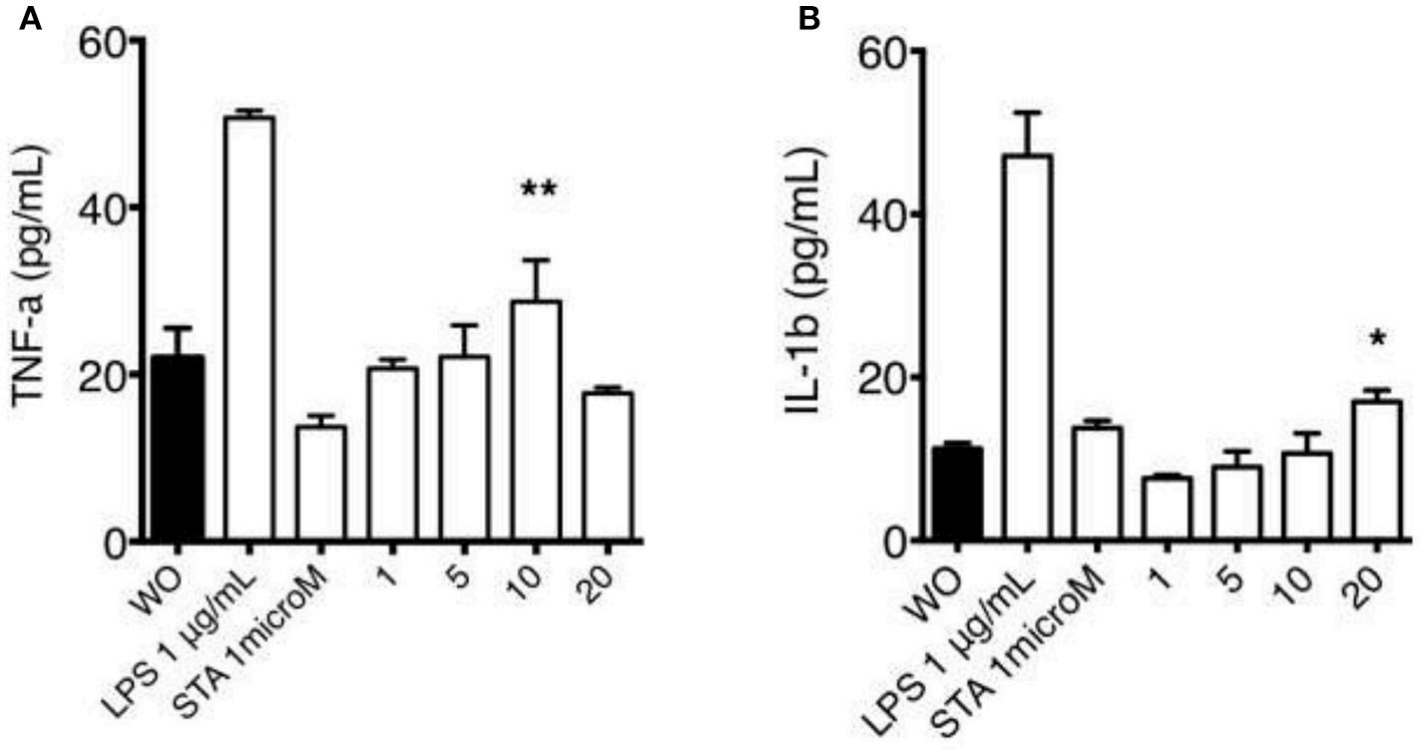
Macrophage secretion of pro-inflammatory cytokines induced by organic extracts exposure. Concentrations of TNF-α **(A)** and IL-1β **(B)** were measured by ELISA in culture supernatant from THP-1 macrophages exposed to organic extracts (1, 5, and 10 μg/ml) for 24 h. Bars represent the mean ± SD form ten independent experiments. Cells stimulated with 1 μM staurosporine and 1 μg/ml LPS (Sigma-Aldrich) were included as a positive apoptosis control. Mean ± SD are represented form 10 independent experiments. The statistical analyses were performed using ANOVA and Dunnett's *post-hoc* test compared to untreated macrophages (black bar). ^**^P < 0.01, ^*^P < 0.05.

We next sought to determine if THP-1 macrophages exposed to organic extracts for 24 h were able to secrete proinflammatory cytokines as IFN-γ, TNF-α, and IL-1β (Figure 9). We did not observe IFN-γ secretion after macrophages were exposed to organic extracts. We found the highest concentration of TNF-α with the 10 μg/ml stimuli (*P* = 0.01) and IL-1β increased in a dose-dependent manner (*P* = 0.05). TNF-α levels that decreased with the higher dose of organic extracts probably due to severe cell damage. These signaling molecules constitute a major factor in the development of airway inflammation.

## Discussion

According to the WHO in 2012 more than 3 billion deaths were attributable to ambient air pollution and more than 80% occur in low- and middle-income countries (12). Most of the people exposed to indoor air pollution usually cook and heat their homes by using solid fuels like wood, dung and crop wastes. Wood combustion produces a very complex mixture of chemical compounds as the carcinogenic PAHs. In the lung, alveolar macrophages and epithelial cells are the central elements of the innate immune system responsible for pathogen and non-pathogen removal.

In this study, we provided scientific evidence relevant to the detrimental effects that early exposure to PAHs present in WSP has on human macrophages as a possible explanation for the association observed between wood smoke exposure and development of pulmonary tuberculosis. By using an *in vitro* experimental model, we have found that PAHs induce phenotypic and functional changes in the macrophage. Some of these changes modify the ability of the macrophage to restrict intracellular bacterial growth. The organic extraction and analysis of the WSP showed that PAHs were present, and the predominant PAHs found in these emissions agree fairly well with the results from other studies previously published (13, 14). Benzo-a-anthracene, fluoranthene, and benzo-a-fluoranthene were the most predominant compounds, and all of them can induce immunotoxicity (Figure 1) (15). Due to their chemical nature, PAH can diffuse cell membranes, to bind cytosolic receptors, this interaction would be responsible for PAHs effect on cell growth and differentiation.

Human macrophages are able to engulf particles made of carbon black with PAHs adsorbed, a phenomenon that is followed by increased release of proinflammatory cytokine secretion. It has been well-demonstrated that most of the PAHs are metabolized by the P450 system and other cellular pathways leading to the production of molecules binding DNA, inducing mutations and tumor development. Production of reactive oxygen species is also critical for the oxidative and proinflammatory microenvironment in which immune cells are present. However, an important contribution of this study is that we analyzed how PAHs present in WSP affect some of the immune mechanisms that macrophages particularly use to limit the growth of pathogenic *M. tuberculosis*.

Our first approach was to observe how innate receptors changed their expression profile when THP-1 macrophages were pre-exposed to increasing concentrations of organic extracts. Previous toxicology studies using PAHs had been focused on the immunosuppressive effect of these molecules. However, our results showed that PAHs could also activate the immune response (Figure 2). The expression of receptors for cell maturation, activation, and pathogen recognition on the macrophage surface was increased in a dose-dependent manner. When we found that macrophages were matured and activated we speculated that this would be more efficient to control bacterial growth; however, we found that this proinflammatory phenotype was not an immune advantage, instead, the toxic effects of organic extracts were more evident after 4 days and macrophages lost their ability to restrict bacterial growth of *M. tuberculosis* (Figures 3A,B). One possible explanation is that besides the cell maturation and activation, macrophages developed disruption of the mitochondrial membrane potential (Figures 4A,B) making them more prone to cell death as is demonstrated by the annexin V-IP staining (Figures 5A,B). We showed that there is a direct correlation between cell viability reduction and the concentration of organic extracts (Figure 6). WSP have also been reported to induce ROS production by stimulation of lung epithelial and immune cells (16). The inflammatory potential of the organic extracts was analyzed in this study. Our results demonstrated that organic extracts induce ROS production and had a similar inflammatory potential than other molecules as traffic-particles than have been previously studied (Figures 7A–C) (17–19).

Even when the percentage of cell death was not dramatically high, it was present in a dose-dependent manner, and we can speculate that metabolism and cell function were very deteriorated reducing the macrophage ability to elicit antibacterial mechanisms important to control the bacterial growth of *M. tuberculosis*. Another mechanism that is very important for the cell to be able to activate cell defense programs is the production of ATP. We found that the intracellular levels of ATP were almost depleted in a dose-dependent manner while a high concentration of extracellular ATP was measured in the culture supernatant. This high concentration of ATP might have led to the depletion of cytosolic K+ and NLRP3-dependent caspase-1 activation and IL-1β secretion (Figure 8). The IL-1β secretion although not very high was also induced in a dose-dependent manner. TNF-α production followed a similar kinetics (Figure 9).

Our study has some limitations that must be described. First, by using this *in vitro* experimental model, we were unable to analyze the chronic effects that organic extracts could induce in macrophages. Second, wood-smoke particles may have other chemical compounds than PAHs than can participate in the inflammatory immune response. Third, we worked with MDMs but, using alveolar macrophages could provide other interesting data.

In conclusion, we have known for many years that PAHs and other volatile compounds are toxic and can lead to chronic pulmonary and cardiovascular diseases, immune impairment and adverse outcomes. By using a simple *in vitro* experimental model, we demonstrated that macrophage exposure to PAHs present in the organic fraction from the WSP induced phenotypic and functional changes that are important to fight against intracellular pathogens like *M. tuberculosis*. It is probably that the concentrations of organic extracts we used were above those observed indoors; however, the cumulative exposure (time and concentration) of the very young (under 5 years old) and women must be similar, and the immune impairment remains over time making them more susceptible to pulmonary tuberculosis as well as other infectious diseases of the lung.

Our results show that phenotype, metabolic and oxidative stress as well as pro-inflammatory responses of macrophages significantly differ after an early exposure to PAHs. These observations confirm the hypothesis that PAHs derived from wood smoke, constitute an important factor in macrophage toxicity. These immunological changes have a direct impact in pulmonary health. Within the possibility of further studies, the spectrum of sampling sites in Mexico, particle sources, their size and toxicological analysis should be extended for a better and comprehensive understanding of the association between cause (acute and chronic exposure) and the immune-toxicological effect.

## Author contributions

IS-O conceived, designed the experiments, and wrote the manuscript. LV and LC-G performed some experiments. IR-P purified the PAHs. AR-V, RP-P, and LT-B analyzed the data.

### Conflict of interest statement

The authors declare that the research was conducted in the absence of any commercial or financial relationships that could be construed as a potential conflict of interest.

## Footnotes

^1^http://www.who.int/mediacentre/factsheets/fs292/en/
