# Supplementary material for: Macrophage Exposure to Polycyclic Aromatic Hydrocarbons From Wood Smoke Reduces the Ability to Control Growth of Mycobacterium tuberculosis
Source: Front Med (Lausanne). 2018 Nov 13;5:309. doi: 10.3389/fmed.2018.00309 (PMC6243050; doi:10.3389/fmed.2018.00309)
Supplement: Supplementary file 1 [file Image_1.pdf]

Macrophage exposure to polycyclic aromatic hydrocarbons from wood smoke reduces the ability to control growth of *Mycobacterium tuberculosis*.

Authors:
Isabel Sada-Ovalle[1], Leslie Chávez-Galán[1], Luis Vasquez[1], Stepahnie Aldriguetti[1], Irma Rosas-Perez[3], Alejandra Ramiréz-Venegas[4], Rogelio Perez-Padilla[4], Luis Torre-Bouscoulet[2]*

[1] Laboratorio de Inmunología Integrativa, Instituto Nacional de Enfermedades Respiratorias Ismael Cosío Villegas, Ciudad de México, Mexico.
[2] Medica Sur, Clinic & Foundation, Mexico City.
[3] Departamento de Ciencias Ambientales, Universidad Nacional Autonoma de Mexico, Ciudad de Mexico, Mexico.
[4] Departmento de Tabaquismo, Instituto Nacional de Enfermedades Respiratorias Ismael Cosío Villegas, Ciudad de México, Mexico.

*** Correspondence:**
Luis Torre Bouscoulet M.D.
E-mail: luistorreb@gmail.com

Supplemental Figure 1

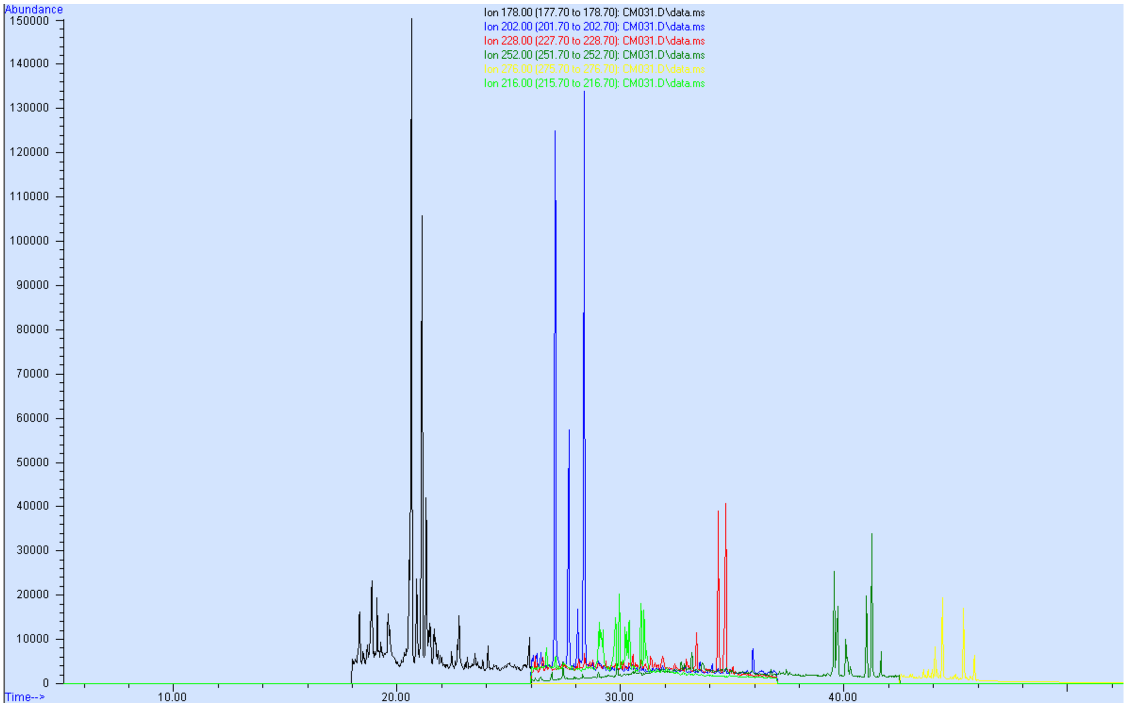

Supplementary Figure 1. Total-ion current chromatogram obtained from GC-MS analysis of a sample of wood smoke (the numbers refer to the substances in Table 1).

Supplementary Table 1

Marker	Clone	Fluorochrome conjugated	Company
CD80	2D10	PE/Cy5	BioLegend
CD86	IT2.2	PE	BioLegend
HLA-DR	L243	APC/CY7	BioLegend
IL-1β receptor	FAB269F	FITC	BioLegend
TNF-α receptor 1	16803	PE	BioLegend
TNF-α receptor 2	22235	APC	BioLegend
TLR-2	TL2.1	AlexaFLuor 647	BioLegend
TLR-4	HTA125	Brillian Violet 421	BioLegend
MMR	15-2	Pe/Cy7	BioLegend
DC-SIGN	9E9A8	FITC	BioLegend
